# Enrichment and characterisation of ethanol chain elongating communities from natural and engineered environments

**DOI:** 10.1038/s41598-020-60052-z

**Published:** 2020-02-28

**Authors:** Pieter Candry, Shengle Huang, José Maria Carvajal-Arroyo, Korneel Rabaey, Ramon Ganigue

**Affiliations:** Center for Microbial Ecology and Technology (CMET), Faculty of Bioscience Engineering, Ghent University, Coupure Links 653, 9000 Gent, Belgium

**Keywords:** Environmental biotechnology, Microbial ecology

## Abstract

Chain elongation is a microbial process in which an electron donor, such as ethanol, is used to elongate short chain carboxylic acids, such as acetic acid, to medium chain carboxylic acids. This metabolism has been extensively investigated, but the spread and differentiation of chain elongators in the environment remains unexplored. Here, chain elongating communities were enriched from several inocula (3 anaerobic digesters, 2 animal faeces and 1 caproic acid producing environment) using ethanol and acetic acid as substrates at pH 7 and 5.5. This approach showed that (i) the inoculum’s origin determines the pH where native chain elongators can grow; (ii) pH affects caproic acid production, with average caproic acid concentrations of 6.4 ± 1.6  g·L^−1^ at pH 7, versus 2.3 ± 1.8  g·L^−1^ at pH 5.5; however (iii) pH does not affect growth rates significantly; (iv) all communities contained a close relative of the known chain elongator *Clostridium kluyveri*; and (v) low pH selects for communities more enriched in this *Clostridium kluyveri*-relative (57.6 ± 23.2% at pH 7, 96.9 ± 1.2% at pH 5.5). These observations show that ethanol-consuming chain elongators can be found in several natural and engineered environments, but are not the same everywhere, emphasising the need for careful inoculum selection during process development.

## Introduction

Carboxylic acids with carbon chains of six to twelve carbon atoms, also named medium chain carboxylic acids (MCCA), are a group of chemicals with a wide range of potential applications; as antimicrobial agents in feed and fodder these chemicals could replace conventional antibiotics, while they can also be used as a corrosion inhibitor^1^. Furthermore, chemical derivatization of MCCA creates additional product opportunities, e.g. esterification of MCCA can lead to a range of fragrance and flavour products for use in cosmetics and food^2^. On top of that, conversion of the carboxylic acids to their relative alcohols and alkanes, can supply solvents and fuels^3^, while Kolbe electrolysis of MCCA allows the production of long chain alkanes from MCCA, for instance decane, usable as jet-fuel^4^.

Caproic acid, the shortest of the MCCA (six-carbon chain), is currently sourced from plant oils and animal fats^5^ with a production capacity of 25,000 tonnes per year and a market value of approx. €2000/tonne^6^. It has generated great research interest because of the aforementioned application potential, in combination with the possibility of sustainable sourcing of caproic acid through bioproduction from waste streams, such as organic wastes and syngas^7–9^. This bioproduction occurs through the so-called reversed-β oxidation pathway, by which an electron donor, such as ethanol, is converted to acetyl-coA. This acetyl-CoA is then used in a cyclical process that converts acetic acid to butyric acid, which is in turn converted to caproic acid. This stepwise two-carbon increase of the carboxylic acid acyl-chain is why this metabolism is often named chain elongation. *Clostridium kluyveri* was the first isolated bacterium capable of producing caproic acid and since then it has been thoroughly investigated, mostly focusing on its unique metabolism^10–13^.

In what concerns the engineering of caproic acid bio-production, most studies have used open communities because they can offer several advantages over axenic cultures: (i) no need for sterility, as the community is functionally stabilized by the operational conditions; (ii) increased process stability under changing conditions, i.e. present but inactive species can take over the role of failing organisms, so-called functional redundancy in open communities^14^; and (iii) in the specific case of chain elongation for production of caproic acid, open communities have been shown to outperform the pure culture *C. kluyveri*^15^. Most studies investigating the use of open communities for ethanol-based caproic acid-production worked at either circumneutral pH or mildly acidic pH (often around 5.5). On a process level, a lower operational pH can be interesting because it inhibits methanogens. Working at reduced pH (and closer to its pKa, 4.88^16^) implies that a larger fraction of the carboxylic acid pool will be present as the undissociated acid. Solvent-based extraction methods for MCCA target this undissociated acid^17^. In this way, working in the vicinity of the pKa of the MCCA can facilitate selective *in situ* extraction methods^18^. On the other hand, the undissociated acids are more toxic than their the dissociated, anionic counterparts, either through import of protons into the cell, or by changing membrane properties and disturbing membrane-associated processes^19–21^. Furthermore, acidic pH are not favourable for growth of known ethanol chain elongators: the type-strain of *C. kluyveri*, strain DSM555, has an optimum pH of 6.4, and grows in a pH range between 6 and 7.5^10,22^. Another, more recent, isolate obtained from bovine rumen – strain 3231B – has been demonstrated to grow at pH as low as 4.88, although the optimal pH for growth of this strain also lies between pH 6.4 and 7.6^23^.

Despite the wide use of open cultures for ethanol chain elongation, the ecology of chain elongating microbiomes is not yet well characterized. Most ethanol-chain elongation studies have been conducted at circumneutral pH, and result in *Clostridium*-dominated communities, often specifically enriched in *C. kluyveri*^24–26^. Only two studies have investigated the community structure at lower pH, both in reactors inoculated from the same system producing caproic acid from diluted corn-to-ethanol beer, and equipped with *in-situ* product extraction^7,27^. In a first study, a reactor coupled to *in situ* product extraction was operated at pH 5.5 for 186 days, fed with synthetic ethanol and acetate mixtures. After 100 days of operation they observed that the community became enriched in *Acinetobacter*, and transitioned by day 160 to domination by *Rhodocyclaceae* neither of which are known chain elongators^7^. The second study used the same operational conditions and same inoculum, but was fed with diluted wine lees waste, leading to a community dominated by OTUs linked to *Ruminococcus, Bacteroides* and *Clostridiales*^27^. These observations show that the community composition and structure can be strongly influenced by operational pH, as well as substrate composition.

In this study the performance and microbial structure of enriched undefined communities producing caproic acid from ethanol and acetic acid was investigated as influenced by the cultivation pH and the source of the inoculum. We hypothesized that the source of inoculum and operational pH could have far-reaching consequences with regards to production rates, production specificities and final community composition, structure and diversity.

## Results

### Production of caproic acid from ethanol & acetic acid by enriched communities

Initially, 6 inocula were enriched at two different pH (7 and 5.5) in duplicate, resulting in 24 enrichment lines. After 3 transfers, only enrichments showing visible growth were retained for further experiments (Supplementary Information, Fig. S.1). This yielded 15 successfully enriched communities: 9 enrichments at pH 7 and 6 enrichments at pH 5.5. All inocula originating from full-scale anaerobic digesters could be enriched at both pH values, the animal faecal inocula could only be enriched at pH 7 and the inoculum taken from the thin stillage fermenter – operated at pH 5.5 – could only be enriched at pH 5.5.

**Figure 1:**
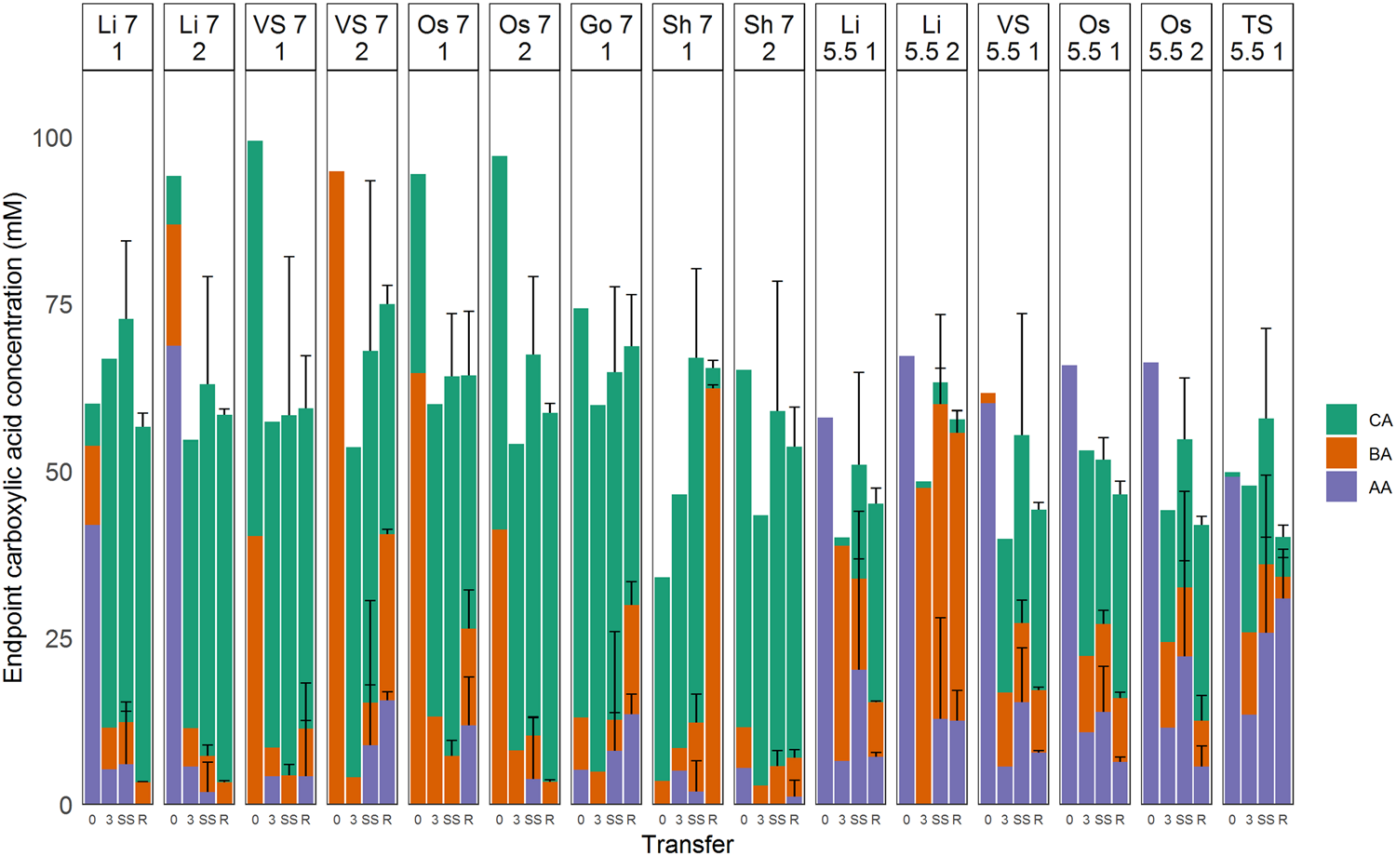
Carboxylic acid concentrations at the end of incubation for each enrichment. Y-axis shows concentrations in mM, x-axis is transfer number per enrichment line. Transfer 0 is the endpoint of the initial transfer, Transfer 3 is endpoint of the third transfer, when non-growing enrichments were dropped. SS is steady-state concentrations, averaging concentrations over transfers 7 to 12 (n = 6). R represents endpoint carboxylate concentrations at the end of the growth curve experiment (n = 4–5). Error bars for SS and R represent standard deviation. Sample names were constructed of inoculum origin, enrichment pH and replicate number, e.g. Li 7 1 represents replicate 1 of Lindemans inoculum enriched at pH 7. Abbreviations: AA = Acetic acid, BA = Butyric acid, CA = Caproic acid.

Most of the eventually successful enrichments at pH 7 produced caproic acid from the first batch (transfer 0), although there was often a notable fraction of butyric acid present as well (Fig. 1). Based on carboxylic acid production, none of their pH 5.5 counterparts were active in this first transfer. Some of these initial enrichments contained more acetic acid than supplemented in the medium, potentially originating from the inoculum, although this was not analysed. By the third generation, when non-growing cultures were dropped, almost all cultures had a product spectrum dominated by caproic acid, the only exceptions being enrichments from the Lindemans anaerobic digester at pH 5.5, which produced butyric acid but no caproic acid. At that timepoint, all enrichments at pH 7 produced more caproic acid (5.5 ± 0.7  g caproic acid.L^−1^, n = 9) than enrichments at pH 5.5 (1.9 ± 1.4  g caproic acid.L^−1^, n = 6). After 12 transfers, an arbitrary steady-state period was defined over 6 transfers (transfers 6–12) for calculation of steady-state concentration profiles. Enrichments at pH 7 again consistently resulted in higher concentrations of caproic acid than those at pH 5.5 (6.4 ± 1.6  g caproic acid.L^−1^ (n = 54) vs. 2.3 ± 1.8  g caproic acid.L^−1^ (n = 36)). The pH remained relatively stable during incubations, with final pH of 6.44 ± 0.21 for enrichments at pH 7, and 5.53 ± 0.10 for enrichments at pH 5.5. Throughout this six-transfer steady-state period, only Li 5.5-1 showed changes in its performance, gradually transitioning from production of butyric acid to caproic acid (explaining the high standard deviations), whereas the second enrichment of this inoculum (Li 5.5-2) only produced butyric acid throughout the entire study.

Cultures were stored at 4 °C and later revived for the determination of growth rates of these enrichments. The average product profile (n = 3–5) at the end of the growth curve experiment shows that in some cases the long-term storage of the cultures influenced their ability to produce caproic acid. Enrichments VS 7-2, Os 7-1, Go 7-1 saw a reduction of caproic acid and uptick of butyric acid concentrations, but caproic acid remained the dominant product. Only in enrichment Sh 7-1 did butyric acid become the dominant product after revival from storage, taking up 95.4 ± 1.9% (molar basis) of the product spectrum. Additionally, enrichment TS 5.5-1 still showed growth, but production of caproic acid dropped, and biomass formed aggregates rather than grow in suspension (Supplementary Information, Fig. S.2).

### Growth rates of enriched caproic acid-producing communities

**Figure 2:**
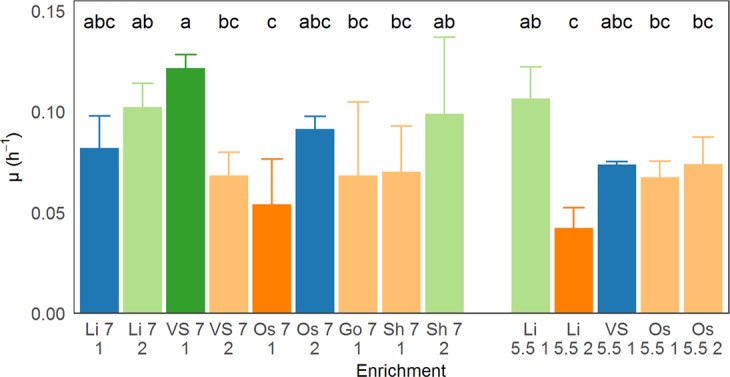
Average growth rate (µ, in h^−1^) of enrichments in the growth rate experiment. Only experiments that showed growth (n = 3–5) were retained. Error bars indicate standard deviation over retained replicates. Colours and labels indicate grouping of enrichments based on Tukey’s HSD test (family-wise confidence level of 0.95); group a (dark green) is significantly different from groups b, c and bc, group ab (light green) is significantly different from group c. Likewise for groups abc (dark blue), bc (light orange) and c (dark orange). Sample names were constructed of inoculum origin, enrichment pH and replicate number, e.g. Li 7 1 represents replicate 1 of Lindemans inoculum enriched at pH 7.

To test whether different operational pH and different inocula affect the kinetics of the chain elongating communities, the growth rate – used as a proxy for production kinetics – of each enrichment was determined in a growth curve experiment in Balch tubes (Fig. 2). Only in enrichment TS 5.5-1 could the growth rate not be determined, due to formation of aggregates after growth (Supplementary Information, Fig. S.2). The highest growth rate was achieved in enrichment VS 7-1 (0.12 ± 0.01  h^−1^), while Li 5.5-2 grew the slowest (0.04 ± 0.01  h^−1^). Not all enrichments at one pH grew at the same rate: while VS 7-1 grew the fastest, Os 7-1 was the slowest enrichment at neutral pH (0.05 ± 0.02  h^−1^). Additionally, variation between enrichments can be very large, for instance, growth rates were significantly different between enrichment VS7-1 and VS7-2, as well as Li 5.5-1 (0.11 ± 0.01  h^−1^) and Li 5.5-2. However, no significant differences (p = 0.19) could be detected between growth rates at pH 7 (0.08 ± 0.03  h^−1^, n = 42) and pH 5.5 (0.07 ± 0.02  h^−1^, n = 21). While these growth rates are in the same order of magnitude as strain 3231B (0.11 h^−1^), the growth rate of strain DSM555 is 2 to 3 times higher (0.24 ± 0.01  h^−1^)^23,28^.

### Community analysis

**Figure 3:**
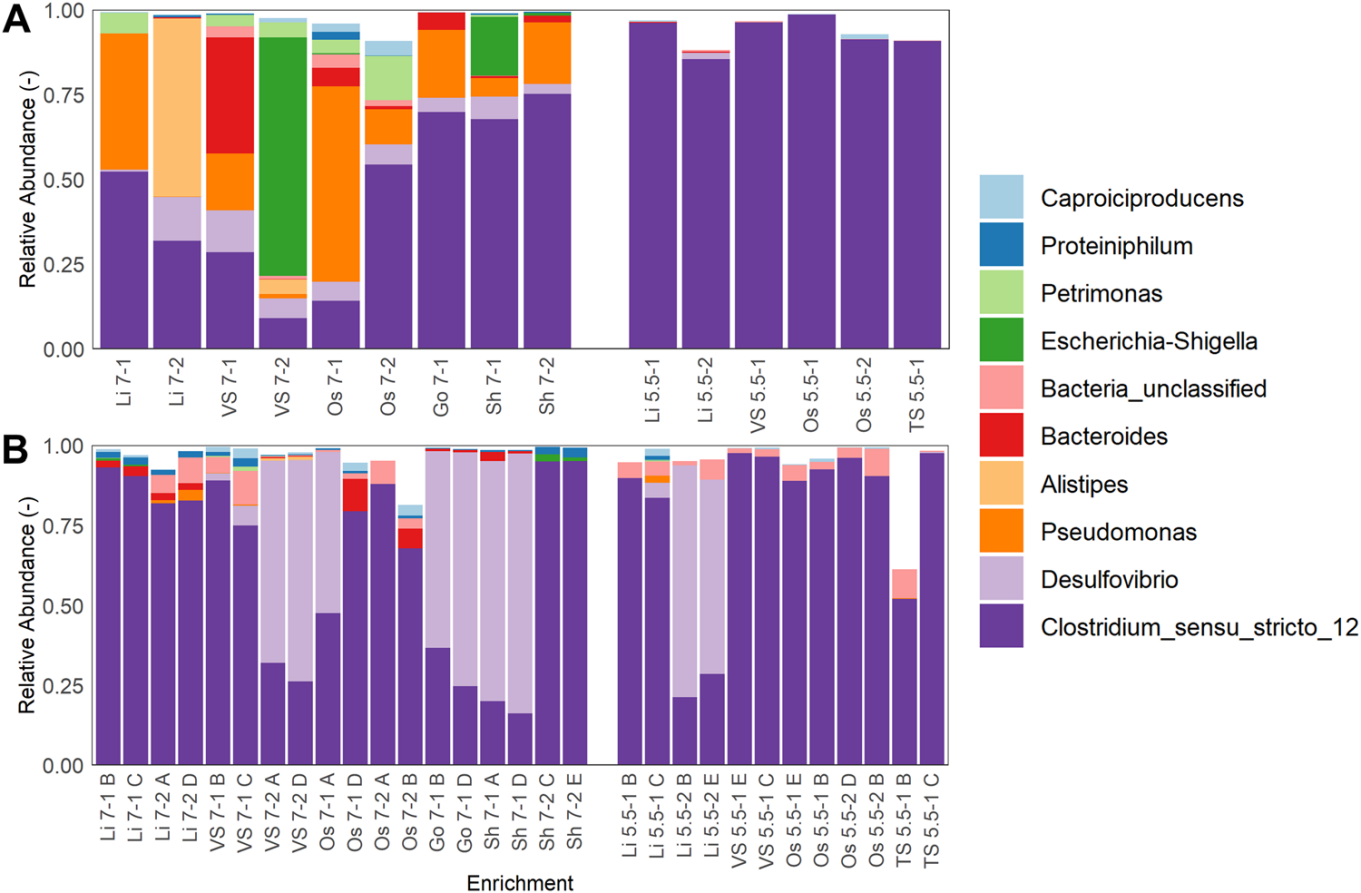
Community composition determined by high throughput amplicon sequencing of enrichment communities. Panel A shows those communities used for flow cytometric fingerprinting (transfer 9 for enrichments at pH 7, transfer 13 or enrichments at pH 5.5). Panel B shows the communities of two randomly selected replicate communities (indicated by letter A-E) from the growth curve experiment after storage. Only the 10 most abundant genera across all samples are shown. The OTU related to C. kluyveri is part of the Clostridium_sensu_stricto_12 group, with all other OTU classified as Clostridium_sensu_stricto_12 amounting to, at most, 0.13% of the total relative abundance (VS 5.5-1). Sample names were constructed of inoculum origin, enrichment pH and replicate number, e.g. Li 7 1 represents replicate 1 of Lindemans inoculum enriched at pH 7.

Two tools were used to characterise the enrichments on a community level: 16S rRNA gene amplicon sequencing and flow cytometric fingerprinting. Amplicon sequencing showed that one operational taxonomic unit (OTU), OTU1, was present in all enrichments at abundances between 9 and 99% of the total community. This OTU was classified as Clostidium_sensu_stricto_12 and represented over 99.87% of all OTUs classified as Clostidium_sensu_stricto_12. Further identification of this organism using the consensus sequence in a BLAST search showed a 100% match with *C. kluyveri* DSM555 and *C. kluyveri* 3231B, with the next closest relative being *C. luticellari* FW341 at 96.8% identity. For this reason, the further text will refer to OTU1 as *C. kluyveri*. Enrichments at pH 5.5 on average contained 96.9 ± 1.2% (n = 6) of *C. kluyveri*, while at pH 7 this was 57.6 ± 23.2% (n = 9) (Fig. 3A). These values show that a large satellite community is present in enrichments at pH 7, and the relative abundance of the 10 most abundant genera shows that this satellite community is enrichment-dependent (Fig. 3). Genera detected in the majority of satellite communities at pH 7 are *Pseudomonas*, *Desulfovibrio, Bacteroides* and *Petrimonas*. Some genera were only detected in a few of the enrichments, such as *Escherischia-Shigella* (VS 7-2 and Sh 7-1) and *Alistipes* (Li 7-2 and VS 7-2).

Storage influenced the community composition in part of the enrichments (Fig. 3B). A first change was an increase in the relative abundance of an OTU closely linked to *Desulfovibrio*, a genus of sulfate-reducing bacteria^29^. This was most noticeable in enrichments VS 7-2, Go 7-1, Sh 7-1 and Li 5.5-2, where *Desulfovibrio* became the most dominant genus, accounting for up to 80% of the community. It should be pointed out that these were the same enrichments that saw an increase in concentrations of butyric acid after storage. A second shift was an increase in the relative abundance of *C. kluyveri* in communities lacking *Desulfovibrio*. In samples that were already highly enriched in *C. kluyveri*, and devoid of *Desulfovibrio*, communities stayed more or less stable. Ultimately, storage either increased the enrichment of the community, or allowed invasion of sulfate reducing bacteria, despite the medium containing low sulfate concentrations (19.5  mg.L^−1^, or 0.2  mM).

**Figure 4:**
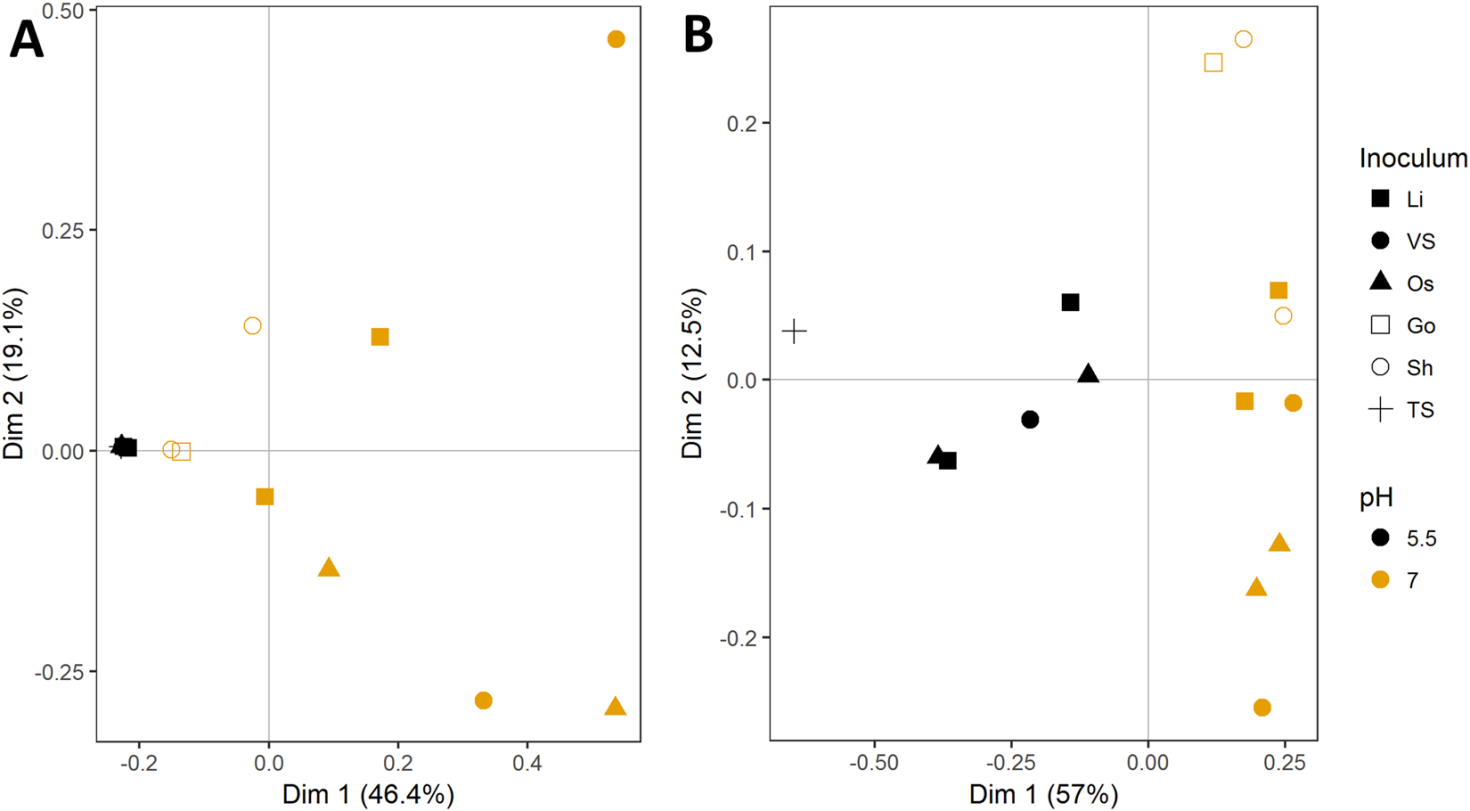
Sequencing and flow cytometry-based comparison of enrichments. Panel A shows community characterization by high-throughput amplicon sequencing, while panel B shows flow cytometric characterization of enriched communities using flow cytometric fingerprinting. Both panels show the principle coordinate analysis (PCoA) based on the Bray-Curtis distance metric between samples. pH and inoculum are indicated by color and shape of the points respectively. Black symbols represent communities enriched at pH 5.5, gold points show those enriched at pH 7. Symbol shapes refer to the initial inoculum the community was enriched from; full square for Lindemans brewery full-scale UASB (Li, ▪), full circle for Van Steenberghe brewery full-scale UASB (VS, •) full triangle for Ossemeersen full-scale waste activated sludge digester (Os, ▲), empty square for goat feces (Go, ▫), empty circle for sheep feces (Sh, ◦), and plus sign for + lab-scale pilot fermenting thin stillage to caproic acid.

To test the hypothesis of diverging communities at different pH, beta-diversity of the community was analysed with taxonomic and flow cytometric data. However, due to the large variation in community structure on the taxonomy level at pH 7, compared to the highly similar communities obtained at pH 5.5, the data was heteroscedastic, preventing further statistical analysis. Amplicon sequencing data was used to build a principle coordinate analysis (PCoA) plot (Fig. 4A), showing a clear separation between samples enriched at pH 7 and pH 5.5. This again confirms the earlier observation of heteroscedasticity, with close clustering of samples at pH 5.5, linked to the high degree of enrichment in *C. kluvyeri*, while samples at pH 7 are more spread out. Alternatively, flow cytometric fingerprinting of the enrichments showed a clear distinction when looking at beta-diversity. Enrichments at pH 7 and pH 5.5 were significantly different (p < 0.01), with pH explaining 55.2% of variation between samples, an analysis that was further confirmed by PCoA analysis of the data, showing clear clustering of enrichments at pH 5.5 vs. pH 7 (Fig. 4B). Lastly, due to the observed shifts in community after storage, it was no longer possible to distinguish between communities grown at pH 7 and pH 5.5 (Supplementary Information, Fig. S.3).

## Discussion

### Inoculum origin determines the operational niche of ethanol chain-elongating communities

Fifteen enriched cultures able to perform chain elongation with ethanol as electron donor were obtained from 6 sources (3 full-scale AD plant, 2 types of animal faeces, and 1 lab-scale pilot installation fed on thin stillage). Under the applied conditions – ethanol and acetate as sole electron donors/acceptors, low sulfate concentrations (0.2  mM) and BES addition to inhibit methanogenesis – chain elongation is the only known thermodynamically feasible metabolism for microbial growth^30^. These selective conditions allowed for a clear investigation on the effect of pH and source of inoculum on the features of chain-elongating microorganisms. The 3 AD-inocula could be enriched successfully at both neutral and mildly acidic pH. On the contrary, samples from animal faeces could only be enriched at neutral pH, while the inoculum from the thin stillage fermenter (pH 5.5) could only be enriched at pH 5.5. These pass-fail results show ethanol-based chain elongators are widely present, but the environment dictates the operational niche of the chain elongators. The pH of the intestinal tract of goat and sheep are close to neutrality, with a pH of 6.2–6.3^31^. This environment could select against micro-organisms that are able to thrive during batch cultivation  under mildly acidic conditions (pH 5.5), as performed in this study. Similarly, the thin stillage fermenter was run in CSTR mode, selecting for micro-organisms tolerant to the combination of low pH and caproic acid. Interestingly, these organisms were not able to cope with more lenient conditions at higher pH. Lastly, chain elongators could be enriched at neutral and mildly acidic pH both from the brewery AD granules (Lindemans, Van Steenberghe) and CSTR sludge AD plant. It is well known that within anaerobic digestion granules, gradients in pH exist^32^. This, in combination with varying feedstock compositions, could either create spatio-temporal niches in anaerobic digesters for chain elongators tolerating lower or higher pH, or select for a chain elongating community that has a broader pH range for growth. However, with the current information – amplicon sequencing and functional data – it is not possible to discern between these two hypotheses. Although this is outside the scope of this study, further investigating these hypotheses could teach us more about the ecological niche of *C. kluyveri* and how it survives and thrives in natural and engineered systems.

The enrichment of one *C. kluyveri*-related OTU that exhibits differential pH preferences suggests that different, currently undescribed *C. kluyveri* strains (or other, closely related species) with varying pH-preferences may have been enriched from the different inocula. To date, two strains of *C. kluyveri* have been isolated, with different functional pH ranges: type-strain DSM555 grows between pH 6 and 7.5 with optimum growth at 6.8, strain 3231B grows between pH 4.9 and 9.1, with optimal growth at pH between 6.4 and 7.6^22,23^. The hypothesis that different *C. kluyveri* strains have different optimal pH and different pH ranges for growth, and are bound to specific pH-influenced niches in the environment could only be confirmed through further research, for instance through isolation of these strains, whole-genome sequencing, etc. The fact that some inocula could only be enriched at one pH indicates that, at least in those cases, this pH window for growth may be narrower than previously observed. Lastly, it should be mentioned that *C. kluyveri* was detected in only one of the six inocula (Go, 0.46% of the community, Supplementary Information, Section S.4). It is highly likely that concentrations in the other inocula were below the detection limit of Illumina-sequencing (0.003–0.01% in the analysed samples). *C. kluyveri* 3231B was isolated from bovine rumen, where the initial concentration of *C. kluyveri* was determined to be 0.00002–0.0002% of the ruminal community^23^. However, as mentioned before, under the imposed, highly restrictive conditions in this study, ethanol chain elongation is the only feasible metabolism, allowing for such high levels of enrichment.

### pH determines product yield and community structure, but not growth rates

Final product concentrations of each enrichment after 12 transfers showed that pH was the key determining parameter affecting caproic acid production, with concentrations in enrichments at pH 5.5 being on average less than half of those at pH 7. The lower concentrations at pH 5.5, with same initial substrate concentrations, indicate toxicity prevented further accumulation, although toxicity of acetic and butyric acids could also play a role. At pH 7 acetate was usually depleted, implying it is likely higher concentrations of caproic acid could have been produced by these communities, if substrate availability had not been limiting. Despite the same substrate provided, the lower final concentrations at pH 5.5 seem to indicate that product toxicity or pH inhibition prevented further increases in product concentration. Although this was not tested in this study, caproic acid is known to be toxic to bacteria at all pH, although lower pH increase this effect^19,28^. While this study was restricted to batch growth of caproic acid-producing communities, *in situ* product extraction could alleviate product toxicity and allow the same conversion efficiency for communities at pH 5.5 and pH 7.

Both flow cytometric and phylogenetic characterisation of the enriched communities showed a clear distinction between communities at pH 5.5 and pH 7. While differences in flow cytometric fingerprints cannot be traced back to specific community shifts, genotypic characterisation showed that variation between communities was much lower at pH 5.5 than at pH 7, specifically due to the higher degree of enrichment of an OTU identifying closely with *C. kluyveri*. Several hypotheses can be proposed as to why the satellite community is larger at pH 7. Firstly, the combination of mildly acidic pH with relatively high concentrations of caproic acid creates a harsh environment, that suppresses non-tolerant organisms. At pH 7 on the other hand, the environment is less harsh, leaving more space for a satellite community to thrive. An alternative hypothesis is that the satellite community is reliant on necrotrophic growth, i.e. growth of bacteria on dead cell material. Communities were transferred after 7 days, rather than at the end of their exponential growth, and growth curve experiments showed that exponential phase was often over well before the 7-day mark. Cultures grown at neutral pH achieved higher optical densities at the end of the exponential phase, providing necrotrophs with more substrate during the stationary phase (Supplementary Information, Fig. S.12). Additionally, the presence of these satellite communities could be methodological artefacts, due to sampling after 7 days of incubation. Initially lenient conditions could allow a satellite community to flourish, while conditions subsequently become more stringent as caproic acid producers start to grow. Additionally, the length of the stationary phase might lead to changes in relative abundance of caproic acid producers and the satellite community, although predicting how this change influences the community would be tenuous. Ultimately, this could lead to the larger satellite community observed at pH 7. In the end, it seems likely that both hypotheses contribute in some way, with pH and caproic acid toxicity being the overarching drivers for the observed differences in satellite community.

Despite these stark differences in product output and community composition, growth rates were relatively similar across enrichments, ranging from 0.04 to 0.12  h^−1^. Given the lower caproic acid production observed at pH 5.5 it was anticipated that cultures at that pH would display significantly lower growth rates than their pH 7 counterparts, however, no significant differences in growth rates could be detected between communities grown at pH 5.5 and pH 7 (Fig. 2). These similar growth rates in most enrichments indicate that energy generation is not affected by pH, and it is likely product toxicity that prevents reaching higher concentrations of caproic acid and higher biomass densities. This in turn implies that efficient *in situ* product extraction technologies could allow for similar production rates at mildly acidic pH vs. circumneutral pH. Operation at lower pH had the additional advantage that it can inhibit methanogenic activity in the system without the need for expensive chemical inhibition. Lastly, we also demonstrated that lower pH selects for a simpler and more specialised community, minimising carbon losses to non-chain elongating community members. This set of traits – cheaper, more efficient operation with no loss of productivity – makes ethanol chain elongation at mildly acidic pH very attractive for future applications.

### Limitations, shortcomings and prospects

This study demonstrates it is possible to enrich ethanol chain-elongation communities from various environments following a simple and straightforward methodology. All inocula selected in this study contained at least some chain elongating microorganisms, despite not being initially detected by the sequencing method applied here. These enriched communities were further characterised and investigated, but some attention should be given to potential pitfalls of the methodology applied.

All obtained enrichments harboured an OTU closely linked to *C. kluyveri*, in varying degrees of enrichment (9–99% relative abundance). The medium used for enrichment was a minimal medium derived from the DSM52 medium for growth of type strain *C. kluyveri* DSM555. This choice might have created a bias towards *C. kluyveri*-centered communities. Other ethanol-consuming chain elongators might have been present in the inocula, but not enriched due to lack of certain nutrients or trace elements in the medium, or, the used batch-culture approach selected for fast-growing organisms, rather than organisms with high substrate affinity but lower growth rates (i.e. r-strategists vs K-strategists^33^). Furthermore, the inoculum selection is by no means exhaustive, and (other) ethanol-consuming chain elongators can be present in other ecological niches. The initial type strain of *C. kluyveri* was isolated from canal mud^10^ and a latter strain obtained from cow rumen^23^. Recently, Kucek and co-workers reported ethanol-chain elongation communities grown on other synthetic media – with yeast extract – to be dominated by *Acinetobacter* and *Rhodocyclaceae*, not previously associated with ethanol-based chain elongation^7^. Given the above, this study is limited to the ecology of *C. kluyveri*-related chain elongators in the 4 engineered and 2 natural systems investigated. Despite this limitation, a distinct variation in pH differentiation was observed between the *C. kluyveri* communities present in these systems that was previously undescribed.

The obtained communities were stored after 12 generations, and later revived for the determination of their growth rates and genotypic community characterisation. It was observed that community structure was – in some cases – impacted by the storage, while in others, it remained unaffected. Several major trends could be observed in the shifts after storage: (i) it was no longer possible to clearly discern communities growing at pH 5.5 from those growing at pH 7 (Supplementary Information, Fig. S.3), (ii) some cultures became more enriched in the *C. kluyveri*-related OTU, and (iii) some other cultures became more enriched in an OTU related to *Desulfovibrio* (Fig. 3). The presence of this *Desulfovibrio*-OTU was unexpected, due to the low concentrations of sulfate in the medium (0.8  mM). However *Desulfovibrio* has been detected in bio-electrochemical systems where degradation of BES was observed^34^. While this study did not quantify BES degradation, it is possible this same process occurred. In some communities where *Desulfovibrio* became more abundant, a shift in product profile from caproic acid to butyric acid was observed. Specifically, it was observed that at pH 5.5, the presence of *Desulfovibrio* (i.e. relative abundance larger than 5%) significantly affected the butyric acid concentration (p < 0.001), while a similar observation at pH 7 was not possible due to the data not being distributed normally (Supplementary Information, Section S.6). A hypothesis for the effect of *Desulfovibrio* on the product profile could be the consumption of ethanol, or consumption of H_2_, which in turn can stimulate anaerobic ethanol oxidation, both possibilities lowering ethanol availability and shifting the product profile towards shorter acids. Although outside the scope of this study, variations between enrichments, microbial community structure and, consequently, the impact of storage could also be influenced by phage presence^35^. This indicates storage and preservation of mixed microbial communities can strongly impact community structure and composition, and care should be taken when doing so. For instance, in previous studies with a pure culture of *C. kluyveri*, researchers removed sulfate from the medium and substituted cysteine as sulfur source to avoid intrusion of sulfate reducing bacteria^15^. Similarly, the stability of enriched communities during storage could be improved by storing and reviving communities in conditions devoid of sulfate, instead using cysteine. Despite this undesired effect, many cultures preserved their caproic acid-producing capabilities, at the same pre-storage levels.

Another note to make is the lack of information on temporal changes in the product profile and community. In the previous section, temporal variation in conditions were hypothesised to impact community composition, for instance through necrotrophic growth or differential growth patterns. However, growth curves (Supplementary Material, Fig. S.12) could be used as a proxy for temporal changes. There it can be observed that the time when stationary phase is reached is highly variable between enrichments. At the same time, there is no clear link between when the stationary phase is reached and community composition or biomass concentration – e.g. higher initial biomass (as OD). However, OD is not an ideal reflection of cell concentrations, and the effect of, for instance, *Desulfovibrio* on growth of *C. kluyveri* could only be thoroughly elucidated by co-culture experiments.

Ultimately, this study demonstrates the presence of ethanol chain elongating organisms in a broad range of natural and engineered environments. The observation that ethanol chain elongating organisms are native to the sampled environments shows that these organisms can (at least) survive in these environments, if not thrive in them. Ethanol chain-elongators likely occupy a specific niche in these systems that they can exploit even at very low abundances, despite the high level of competition for substrates in most of these systems. Little to no information is available on the environmental niches of these organisms, and how they survive in a natural environment, while their widespread presence implies some role in environmental processes. At a bioprocess level, the results of this study show that the choice of inoculum should be made based on the desired process parameters. An environment that harbours only neutrophilic ethanol chain elongating organisms will not be suitable as a source of inoculum for a process where e.g. *in-situ* solvent-based extraction demands a mildly acidic operational pH.

## Conclusions

It was possible to enrich ethanol chain-elongating communities from six different inocula, including not yet studied environments such as brewery digesters and goat and sheep faeces. Clear differences could be observed between different enrichment lines: enrichments originating from anaerobic digesters could tolerate both neutral and mildly acidic pH, while enrichments from animal faeces were only successful at neutral pH and enrichments from a mildly acidic system producing caproic acid only grew under the same conditions. This indicates that the characteristics of ethanol-consuming caproic acid-producers are determined by the environmental niche they were originally occupying.

There were clear differences in the degree of enrichment between communities at pH 7 and pH 5.5. All enrichments were centred around an OTU related to *C. kluyveri*, with a variable satellite community. Under mildly acidic conditions, this satellite community was on average smaller, with a higher degree of enrichment of *C. kluyveri*. It was possible to statistically distinguish communities grown at pH 7 and pH 5.5 using flow cytometric fingerprinting, while this distinction was not possible using 16S rRNA gene-based fingerprinting, due to heteroscedasticity.

Functionally, enrichments at pH 7 produced on average approx. twice the amount of caproic acid when compared to enrichments at pH 5.5, likely due to toxicity effects of caproic acid. However, growth rates of enrichments at pH 5.5 and pH 7 were similar (in the range of 0.04–0.12  h^−1^, on average), indicating that application of *in situ* extraction technologies could result in similar production rates at both pH.

## Methods

### Source of inocula and enrichment procedure

Six inocula were selected for the enrichment of chain elongating communities; 4 engineered environments and 2 natural environments. Two full-scale upflow anaerobic sludge blanket (UASB) treating brewery waste water were sampled, under the hypothesis that infrequent exposure to ethanol might result in a larger fraction of chain elongating organisms. These systems were denoted Li (Lindemans brewery, Vlezenbeek, Belgium) and VS (Van Steenberge brewery, Ertvelde, Belgium). An additional anaerobic digester treating waste activated sludge (Ossemeersen waste water treatment plant (Os, Ghent, Belgium) was sampled as a low ethanol analogue to the two brewery samples. The last engineered system was a lab-scale pilot installation fermenting thin stillage from a bioethanol plant to caproic acid at pH 5 (TS^36^) and was chosen due to its capacity to produce caproic acid at low pH. Lastly, two natural environments (i.e. the gut microbiome of either goat (Go) or sheep (Sh)) were sampled via faecal material to investigate the presence of these organisms in nature. These were selected for their likeliness to contain ethanol-driven chain elongators, as seen by to the isolation of *Clostridium kluyveri* 3231B from cow rumen, but were yet unexplored in literature. It should be emphasised here that the aim was not to find new environments for chain elongators, but rather, to have a fair comparison between chain elongating organisms from these different environments.

The six inocula were enriched in a synthetic medium, modified from the DSM52 medium for *C. kluyveri*, with 15  mL ethanol·L^−1^ and 2.5  g acetate·L^−1^ (Supplementary Information, Section S.8.1). The changes in this medium in comparison to the original medium are: i) a decrease in ethanol and acetic acid concentrations from the original 20  mL·L^−1^ and 10.0 g K-Ac·L^−1^ respectively, to reduce final caproic acid-concentrations and mitigate potential product inhibition, ii) partial substitution of MgSO_4_.7H_2_O with MgCl_2_ to reduce activity of sulfate-reducing bacteria, iii) removal of yeast extract to restrict carbon sources to ethanol and acetic acid, iv) addition of 25 mM BES to inhibit methanogens, with the intent of focusing more deeply on the chain elongating organisms present in the sampled environments, and, v) addition of 25 mM MES to buffer pH during batch incubations. Each inoculum was enriched in duplicate at pH 7 and pH 5.5, resulting in 24 enrichments (Supplementary Information, Fig. S.15). For the initial enrichment (transfer 0), 0.5  mL of inoculum was added to 40  mL of medium. Subsequent transfers used 10% inoculum (v/v basal medium %). Before the start of incubation, the serum flasks headspace pressure was manually corrected to 101 hPa overpressure (Infield 7 tensiometer, UMS) versus atmospheric pressure, at 20 °C. Samples were incubated on a shaker (r = 120  rpm) in a temperature-controlled room (34 °C). Transfer 0 was incubated for 10 days, subsequent transfers were incubated for 7 days. At the end of each transfer, headspace pressure was measured, headspace composition analysed, and samples taken for DNA, OD, pH, and concentrations of carboxylic acids and ethanol. Headspace composition was analysed by GC-TCD, while carboxylic acids and ethanol were analysed with distinct GC-FID methods (Supplementary Information, Section S.8.3).

At the end of transfer 3, serum flasks not showing any growth (i.e. OD < 0.05) were removed from the enrichment, resulting in 15 actively growing cultures. After transfer 9, communities were analysed for flow cytometric fingerprinting. However, because the cell count and productivity in enrichments at pH 5.5 was too low, flow cytometric fingerprinting of these enrichments was repeated at the end of transfer 13. Samples of transfer 9 for pH 7 communities and transfer 13 for pH 5.5 communities were used for amplicon sequencing to compare both fingerprinting methods, as well as on all samples from transfer 12 to remove the effect of different generations. After transfer 13, the enriched communities were sampled, and subsequently stored at 4 °C until later revival. The communities were stored in the serum flasks obtained after 7 days of incubation at 37 °C.

### Growth curves

The effect of the pH and source of inoculum was evaluated by investigating the product spectrum and the growth rate of the different enrichment cultures. Communities stored at 4 °C were revived in Balch tubes after 5 months of storage. Incubations of Balch tubes were done in a temperature-controlled room at 34 °C, shaken horizontally at 120  rpm, to optimise mixing. After 3 transfers under these conditions, a growth curve was performed in Balch tubes, tracking optical density at 600  nm (OD) in the tubes using a Spectronic 200 spectrophotometer (Thermo Fischer Scientific, Netherlands). OD was measured every 4 h until the stationary phase was reached. Samples where no growth was observed were removed from further analysis of both growth rates (µ) and carboxylic acid production. Growth rates were calculated by fitting a Richards model (Eq. 1) to the data after correction with a blank measurement (i.e. Balch tube before inoculation) and log-transformation of the data, in accordance with Begot *et al*.^37^.

1 $$\mathrm{ln}(\frac{\varDelta OD}{\varDelta O{D}_{{\rm{\min }}}})=A\cdot {(1+\nu \cdot {e}^{1+\nu }\cdot {e}^{\frac{\mu }{A}\cdot {(1+\nu )}^{1+\frac{1}{\nu }}\cdot (\lambda -t)})}^{-\frac{1}{\nu }}$$

Subsequently, quartiles (Q_1_, Q_3_) and interquartile range (IQR) were calculated per enrichment, and strong outliers – defined as being outside Q_1_–3*IQR or Q3 + 3*IQR – were removed. After these steps each enrichment had at least 3 replicates for further analysis. Subsequently, normal distribution of growth rates per enrichment was controlled with the Shapiro-Wilk test (shapiro.test function in R), and homoscedasticity was checked across the groups with the Levene test (leveneTest function in the ‘car’ package in R). Analysis of variance (ANOVA, aov function in R) was performed to test whether average growth rates were significantly different between different enrichments, and Tukey’s Honestly Significant Difference (HSD) test (TukeyHSD function in R) was performed with a family-wise confidence level of 0.95, to analyse which enrichments were significantly different from each other. For comparison of growth rates of enrichments at pH 7 versus pH 5.5, a t-test was performed with data after removal of outliers and verifying normal distribution and homoscedasticity of both groups.

### Community characterisation

The enriched communities were characterised by flow cytometric fingerprinting, and with 16 S rRNA gene amplicon sequencing. Flow cytometric fingerprinting was performed according to Props *et al*.^38^, using a FACSVerse 3000 flow cytometer (BD Biosciences, Erembodegem, Belgium). For 16S rRNA gene amplicon sequencing, samples (2 mL in Micrewtubes®) were centrifuged (5 min at 20817 g), supernatant was removed, and remaining pellets were stored at −20 °C until extraction. DNA was extracted from these pellets according to Vilchez-Vargas *et al*.^39^, without the additional column purification step. Illumina MiSeq® was used for high-throughput amplicon sequencing of the V3-V4 region of the 16 S rRNA gene region in accordance with Andersen *et al*.^40^. Amplicon reads were processed in the Mothur pipeline^41^ in accordance with Props *et al*.^38^, resulting in an OTU table. Before subsequent statistical analysis, absolute singletons were removed from this dataset.

The hypothesis of diverging communities at different pH was tested statistically on the generated sequencing and flow cytometry data in R v3.5.1^42^. Flow cytometry data was processed with the PhenoFlow package^38^, where the raw data from the detectors was first transformed with the asinh-function. Plots were subsequently gated to exclude background events, and data was randomly resampled to the smallest cell obtained cell count of all samples (3154 cells, vs. 9677 as highest cell count). The transformed detector data was normalised between 0 and 1. This dataset was then used to calculate the flow cytometric fingerprint of each sample. For amplicon sequencing data, the OTU table obtained after processing of raw sequencing data through the Mothur pipeline, and removal of absolute singletons was first randomly resampled to the lowest read count in all samples (5353 reads). A mock community with known composition was used as quality control for the sequencing (Supplementary Information, Fig. S.16).

After these pre-processing steps, a Bray-Curtis dissimilarity matrix was made with the vegdist function from the vegan package. Variances between enrichments at pH 7 and pH 5.5 in the multiparametric spaces were compared with the betadisper function from the vegan package to check for homoscedasticity. If the homoscedasticity requirement was fulfilled, a permutational multivariate analysis of variance (PERMANOVA) was run, using the adonis function from the vegan package, to calculate statistically significant differences between enrichments at pH 7 and pH 5.5. The Bray-Curtis dissimilarity matrices were also used for PCoA for visual interpretation of the multidimensional data. For amplicon sequencing data, this was done with the cmdscale function, while flow cytometry data was analysed with the beta_div_fcm function from the PhenoFlow package.

## Supplementary information

Supplementary Info.

## Data Availability

The raw fastq files that served as a basis for the bacterial community analysis were deposited in the National Center for Biotechnology Information (NCBI) database (Accession number PRJNA556976).
