# Supplementary material for: Enrichment and characterisation of ethanol chain elongating communities from natural and engineered environments
Source: Sci Rep. 2020 Feb 28;10:3682. doi: 10.1038/s41598-020-60052-z (PMC7048776; doi:10.1038/s41598-020-60052-z)
Supplement: Supplementary file 1 — Supplementary Info. [file 41598_2020_60052_MOESM1_ESM.pdf]

Supplementary material to: Enrichment and characterisation of ethanol chain elongating communities from natural and engineered environments

Pieter Candry[1], Shengle Huang[1], José Maria Carvajal-Arroyo[1], Korneel Rabaey[1], Ramon Ganigué[1,*]

[1] Center for Microbial Ecology and Technology (CMET), Faculty of Bioscience Engineering, Ghent University, Coupure Links 653, 9000 Gent, Belgium

[*] Correspondence to: Ramon Ganigué, Ghent University; Faculty of Bioscience Engineering; Center for Microbial Ecology and Technology (CMET); Coupure Links 653; B-9000 Gent, Belgium; phone: +32 (0)9 264 59 76; fax: +32 (0)9 264 62 48; E-mail: Ramon.Ganigue@UGent.be; Webpage: www.cmet.UGent.be.

S.1. Net growth of enrichments over different transfers

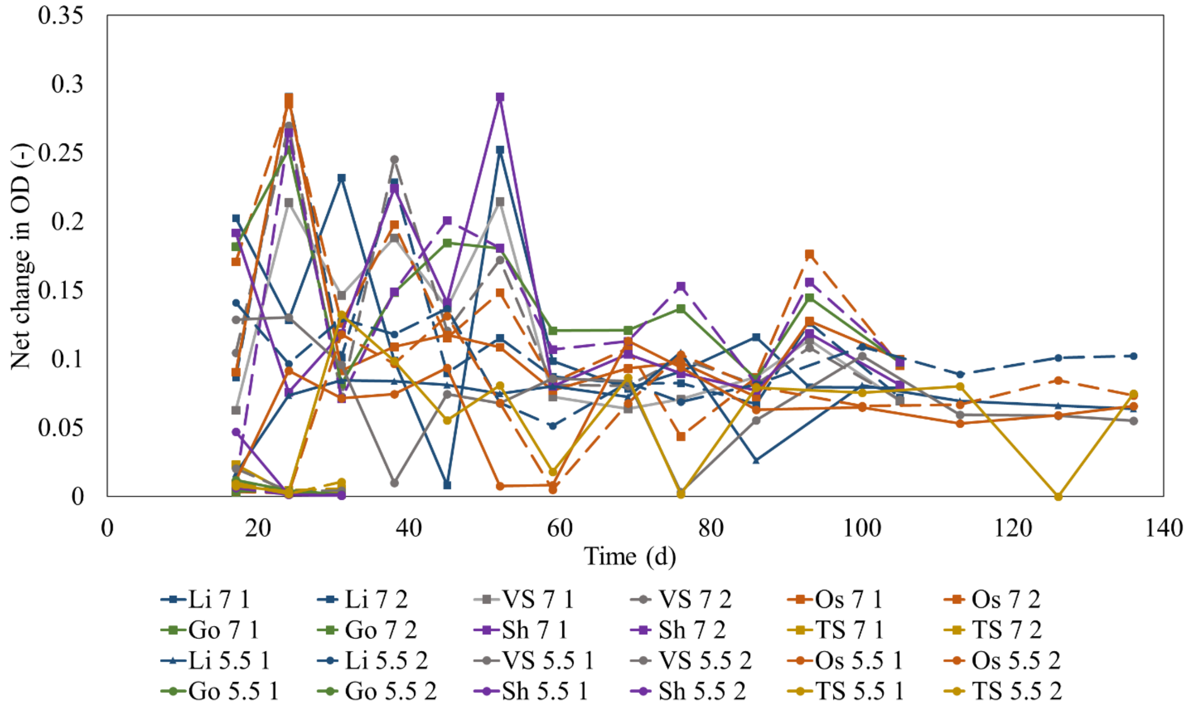

Figure S.1. Net increase of OD at the end of each transfer over time. Colours indicate original inoculum: blue comes from Lindemans brewery full-scale UASB, gray from Van Steenberghe brewery full-scale UASB, orange from Ossemeersen full-scale CSTR AD, green from goat faeces, purple from sheep faeces and golden from thin-stillage lab-scale pilot. Shape of symbols indicates pH of enrichment: squares were enriched at pH 7, circles at pH 5.5. Lines indicate enrichment replicate: full line was replicate 1, dashed line replicate 2. After the third transfer (day 31), all enrichments with an OD below 0.01 were dropped. Sample names were constructed of inoculum origin, enrichment pH and replicate number, e.g. Li 7 1 represents replicate 1 of Lindemans inoculum enriched at pH 7.

S.2. Aggregated growth in enrichment series TS 5.5 1 during growth curves

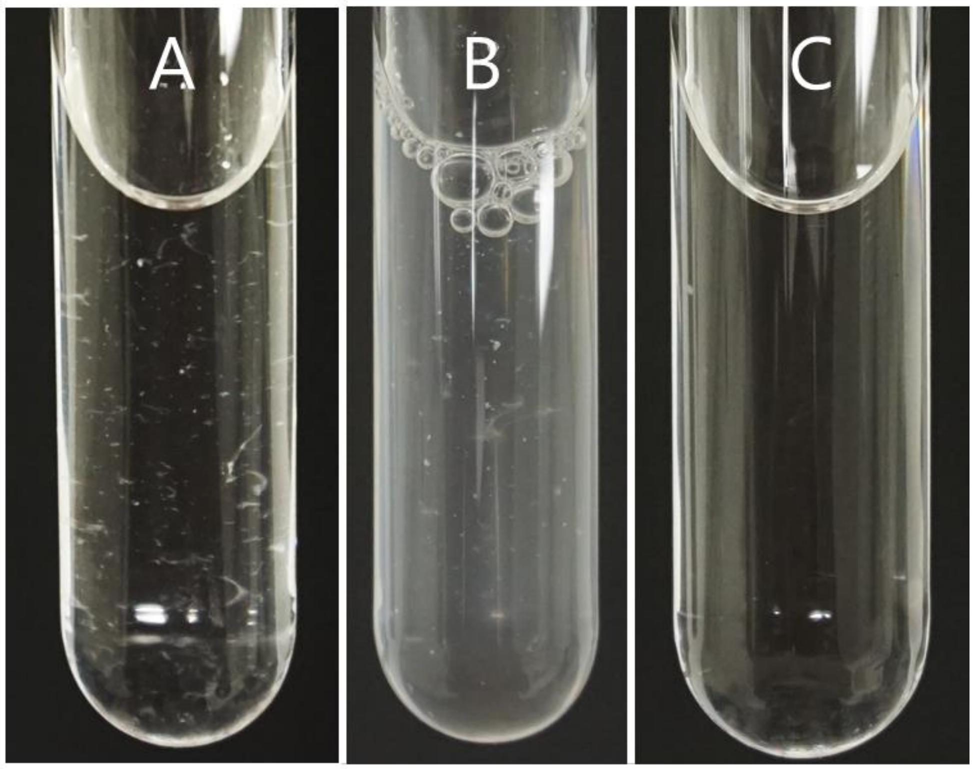

Figure S.2. Pictures of growth in Balch tubes after 7 days of incubation for growth curve experiment. Panel A shows aggregated growth in enrichment TS 5.5 1 (replicate A), Panel B shows suspended growth in Li 5.5 1 (replicate C), and Panel C shows an uninoculated control after 7 days of incubation at 34°C.

S.3. Community composition after enrichments

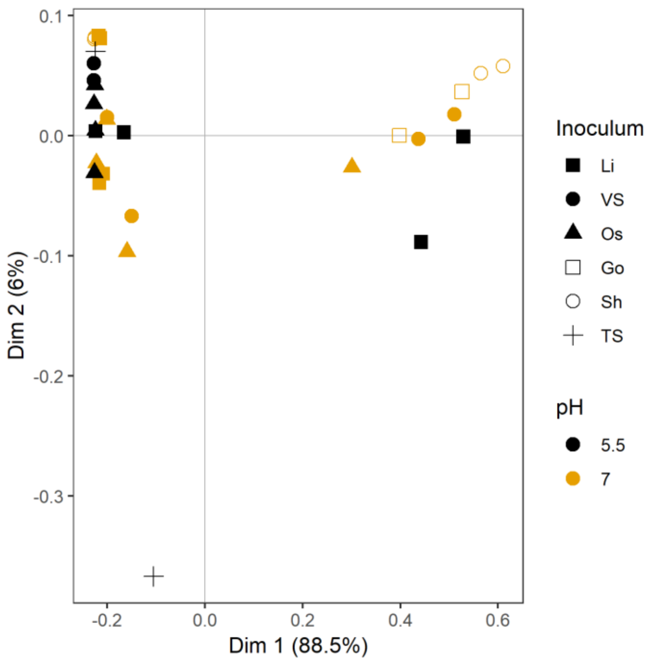

Figure S.3. Beta-diversity analysis of high-throughput amplicon sequencing data (Illumina) of enriched communities after 12 transfers. pH and inoculum are indicated by color and shape of the points respectively. Black symbols represent communities enriched at pH 5.5, gold points those enriched at pH 7. Symbol shapes refer to the initial inoculum the community was enriched from; full square for Lindemans brewery full-scale UASB (Li, ■), full circle for Van Steenberghe brewery full-scale UASB (VS, ●) full triangle for Ossemeersen full-scale waste activated sludge digester (Os, ▲), empty square for goat feces (Go, □), empty circle for sheep feces (Sh, ○), and plus sign for + semi-pilot-scale thin stillage fermenter producing caproic acid.

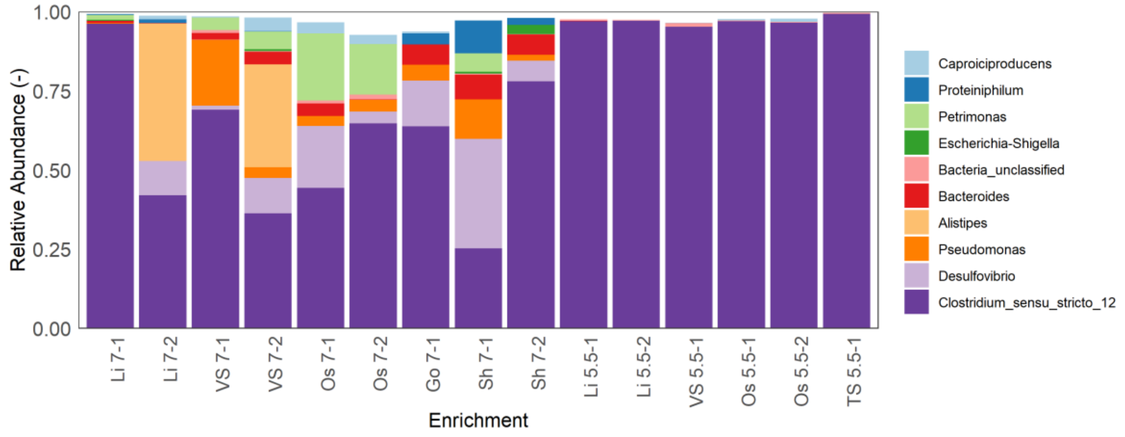

Figure S.4. Community composition determined by high throughput amplicon sequencing of enrichment communities after 12 transfers for all communities Sample names were constructed of inoculum origin, enrichment pH and replicate number, e.g. Li 7 1 represents replicate 1 of Lindemans inoculum enriched at pH 7.

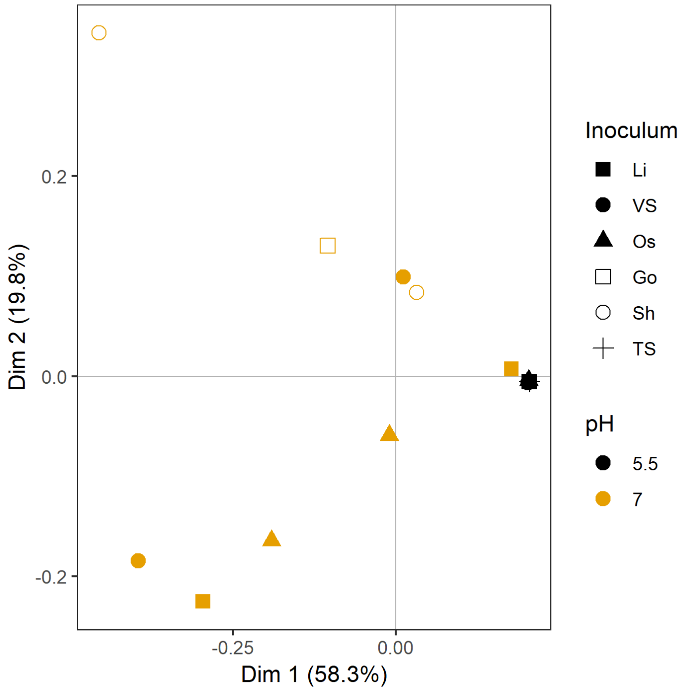

Figure S.5. Beta-diversity analysis of high-throughput amplicon sequencing data (Illumina) of enriched communities after 12 transfers. pH and inoculum are indicated by color and shape of the points respectively. Black symbols represent communities enriched at pH 5.5, gold points those enriched at pH 7. Symbol shapes refer to the initial inoculum the community was enriched from; full square for Lindemans brewery full-scale UASB (Li, ■), full circle for Van Steenberghe brewery full-scale UASB (VS, ●) full triangle for Ossemeersen full-scale waste activated sludge digester (Os, ▲), empty square for goat feces (Go, □), empty circle for sheep feces (Sh, ○), and plus sign for + semi-pilot-scale thin stillage fermenter producing caproic acid.

S.4. Community composition of inocula

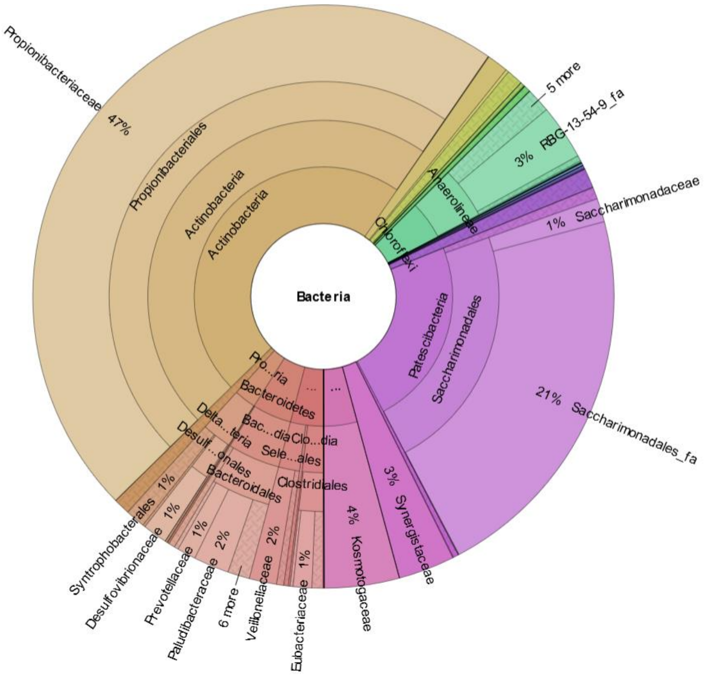

Figure S.6. Krona plot[1] of community presesnt in the full-scale granular sludge digester at Lindemans brewery (Li)

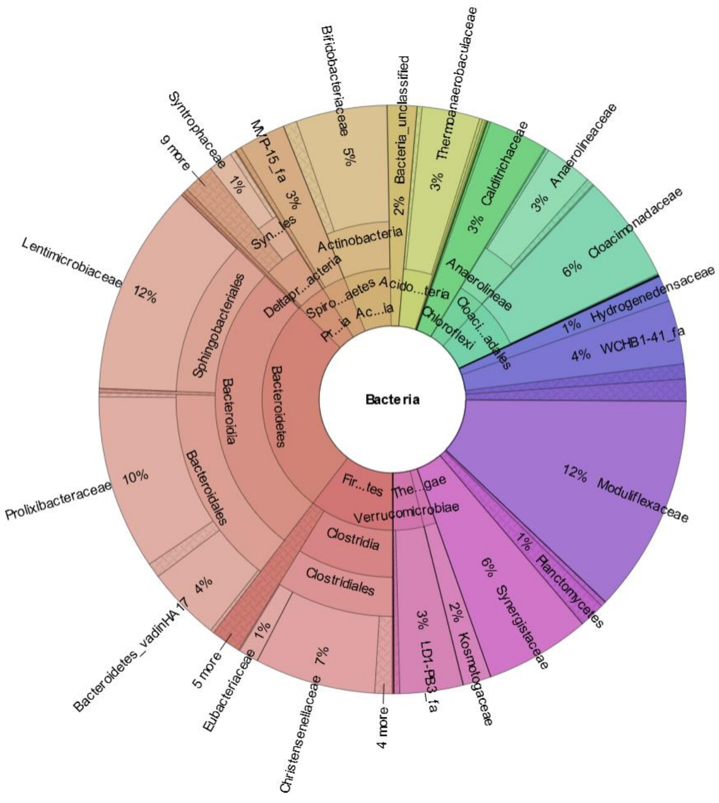

Figure S.7. Krona plot[1] of community present in the full-scale granular sludge digester at Van Steenberge brewery (VS)

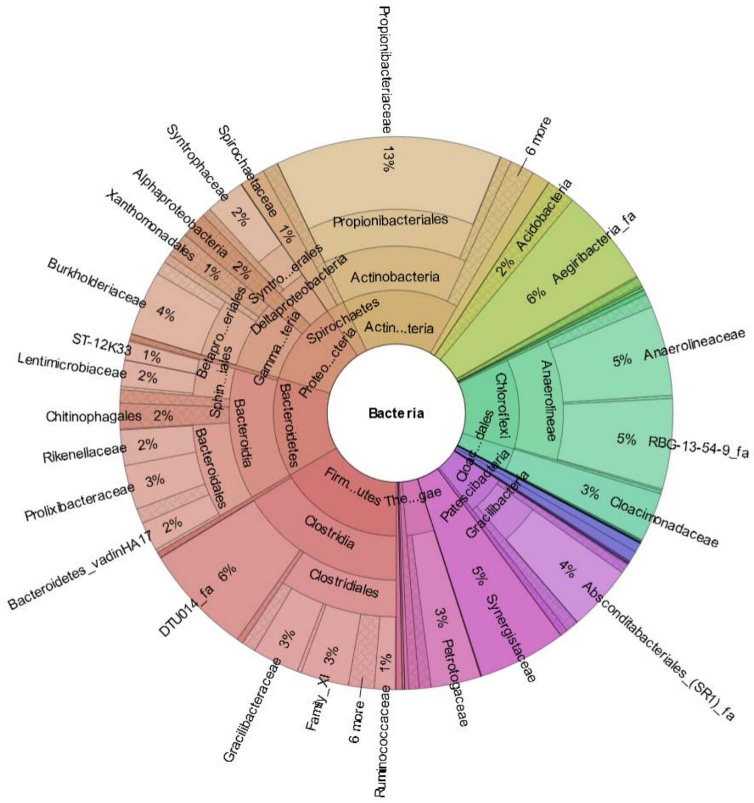

Figure S.8. Krona plot[1] of community presesnt in the full-scale waste activated sludge digester at the Ossemeersen wastewater treatment plant (Os)

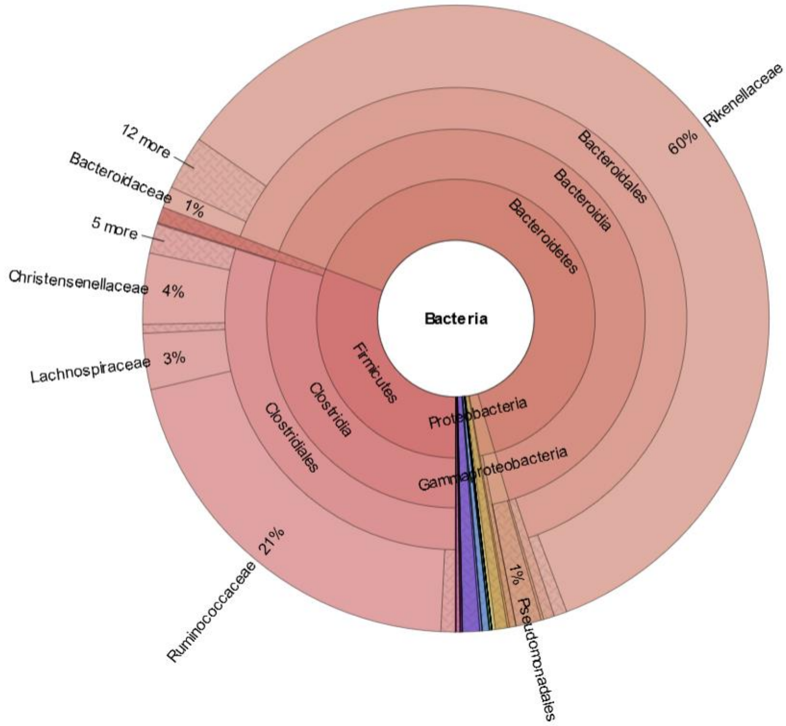

Figure S.9. Krona plot[1] of community present in goat faeces used for this enrichment study (Go)

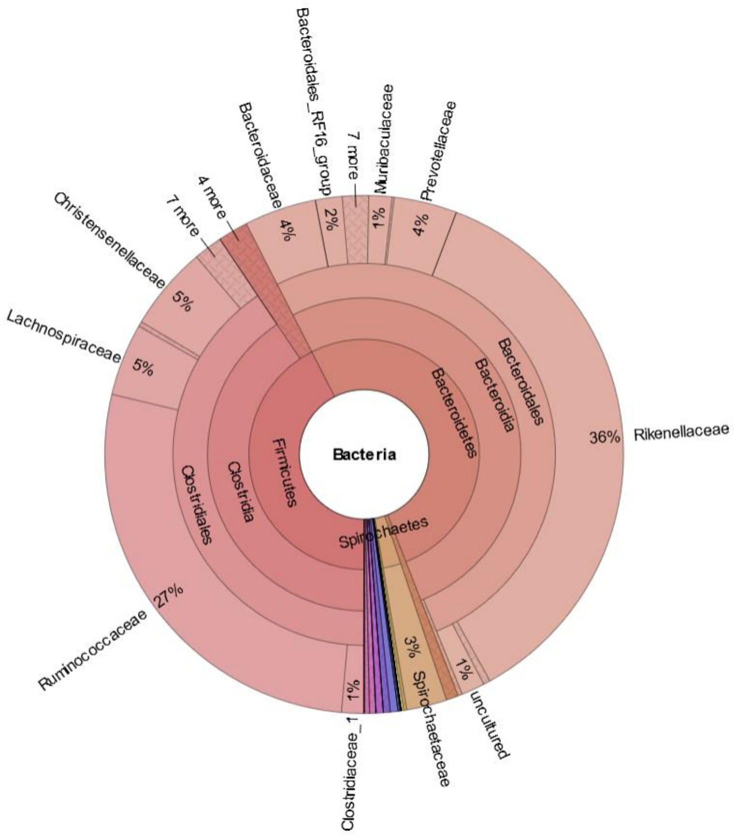

Figure S.10. Krona plot[1] of community present in sheep faeces used for this enrichment study (Sh)

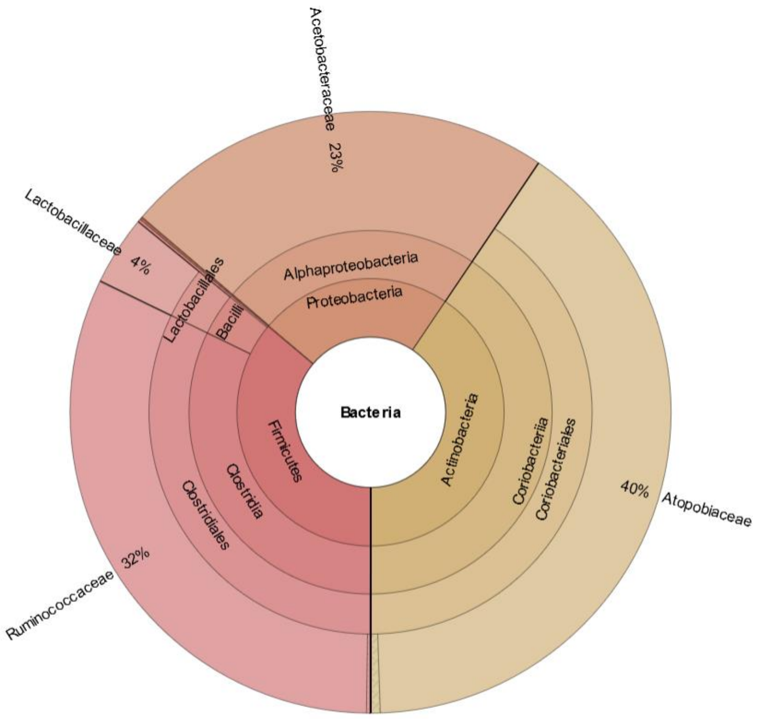

Figure S.11. Krona plot[1] of community present in a thin stillage fermenting lab-scale pilot installation (TS)

S.5. Growth curves for each enrichment series

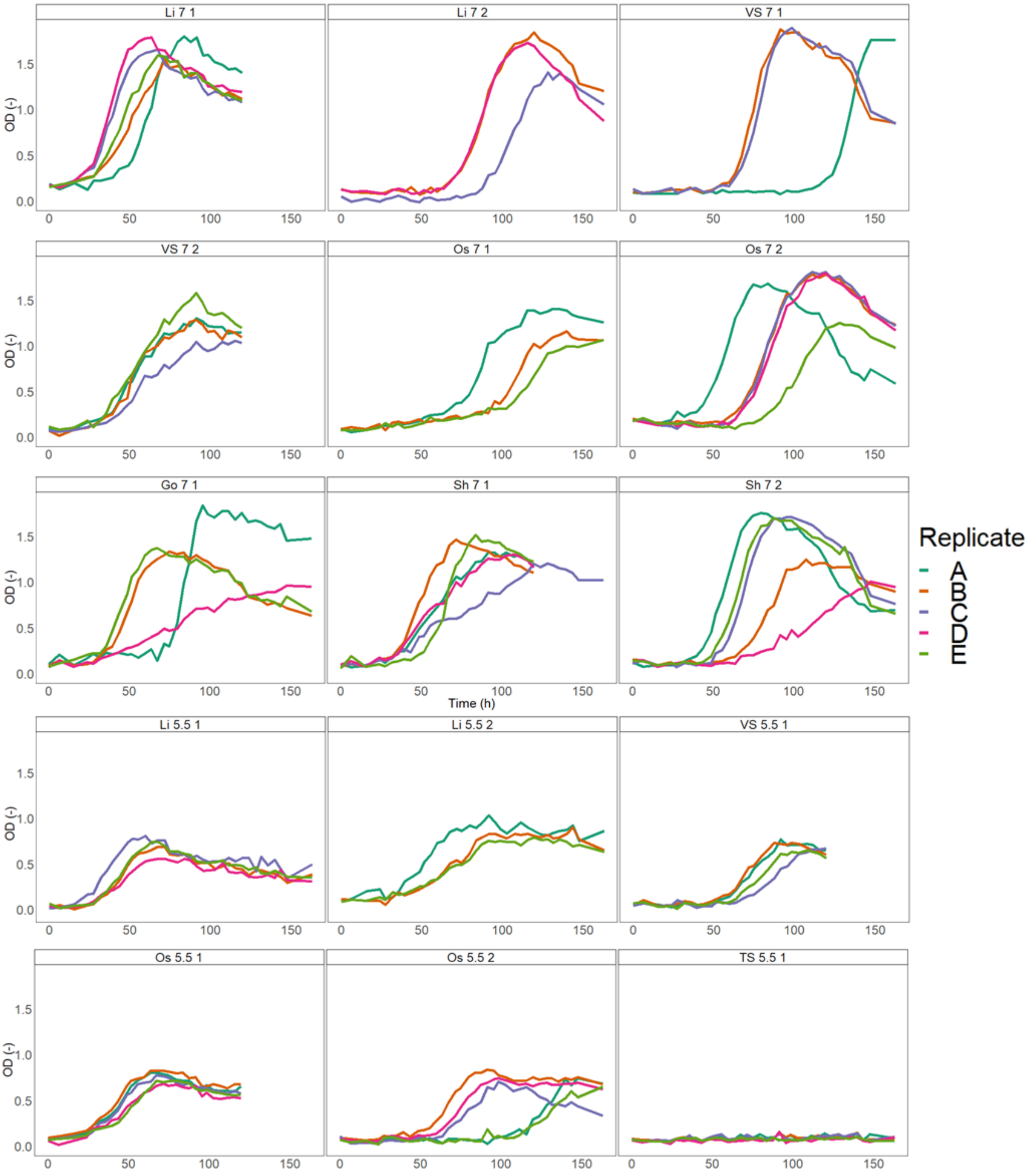

Figure S.12. Growth curves of each enrichment used for further growth rate calculations. In experiments where growth was completed after 120h, measurements were stopped. Sample names were constructed of inoculum origin, enrichment pH and replicate number, e.g. Li 7 1 represents replicate 1 of Lindemans inoculum enriched at pH 7.

S.6. Impact of *Desulfovibrio* on product profile

The impact of the presence of *Desulfovibrio* on the product spectrum was investigated by first looking at the average product profile in communities at pH 7 and pH 5.5 with or without *Desulfovibrio*. The cut-off for presence was arbitrarily put at 5%, to select communities that were highly enriched in *Desulfovibrio*. Looking at the average product profile, both at pH 5.5 and 7 an increase can be seen in butyric acid when *Desulfovibrio* is present, although the spread at pH 7 is quite large (Figure S.13)

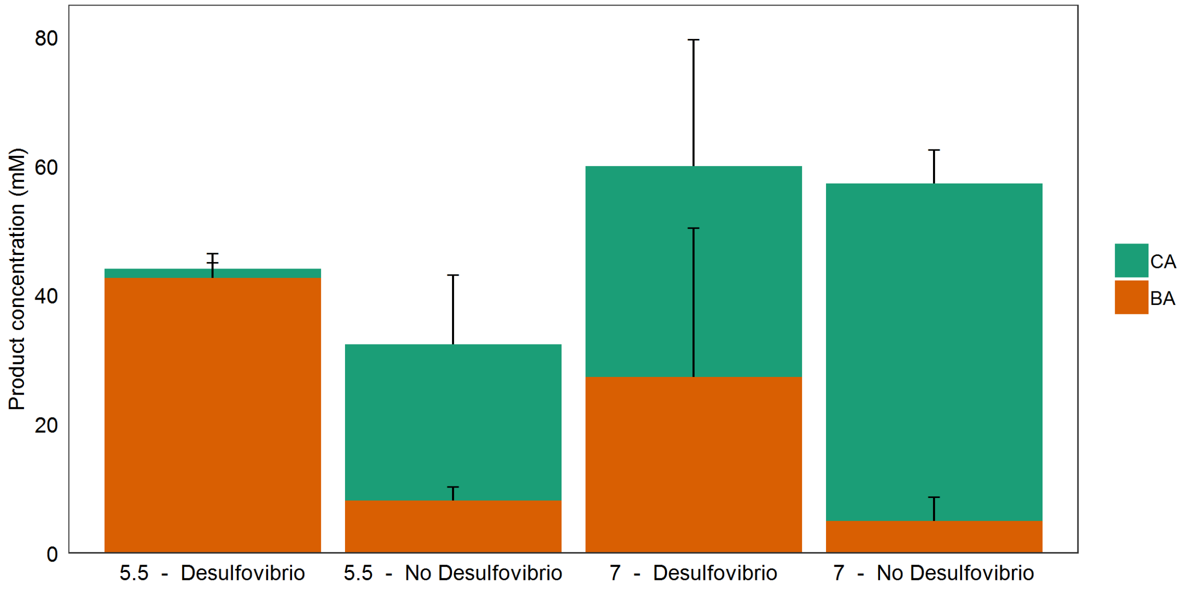

Figure S.13. Average concentrations of butyric acid (BA) and caproic acid (CA) at pH 7 and pH 5.5 after growth curves, comparing communities with *Desulfovibrio* (>5% relative abundance) to those with less than 5% *Desulfovibrio*.

To confirm these observations, an ANOVA test was performed, studying the impact of the presence of *Desulfovibrio* on butyric acid concentrations. Data at pH 5.5 were analysed separately from data at pH 7. While both pH showed a significant p-value for the impact of *Desulfovibrio*-presence ($p=0.008$ for pH 7; $p<0.001$ for pH 5.5), the Shapiro-Wilk normality test showed residuals at pH 7 were not normally distributed ($p=0.001$), making it impossible to make hard claims on this observation at pH 7. We can however conclude that at pH 5.5, the presence of *Desulfovibrio* significantly shifted the product profile towards butyric acid, as residuals were normally distributed ($p=0.19$).

S.7. Electron balance over growth curve experiment

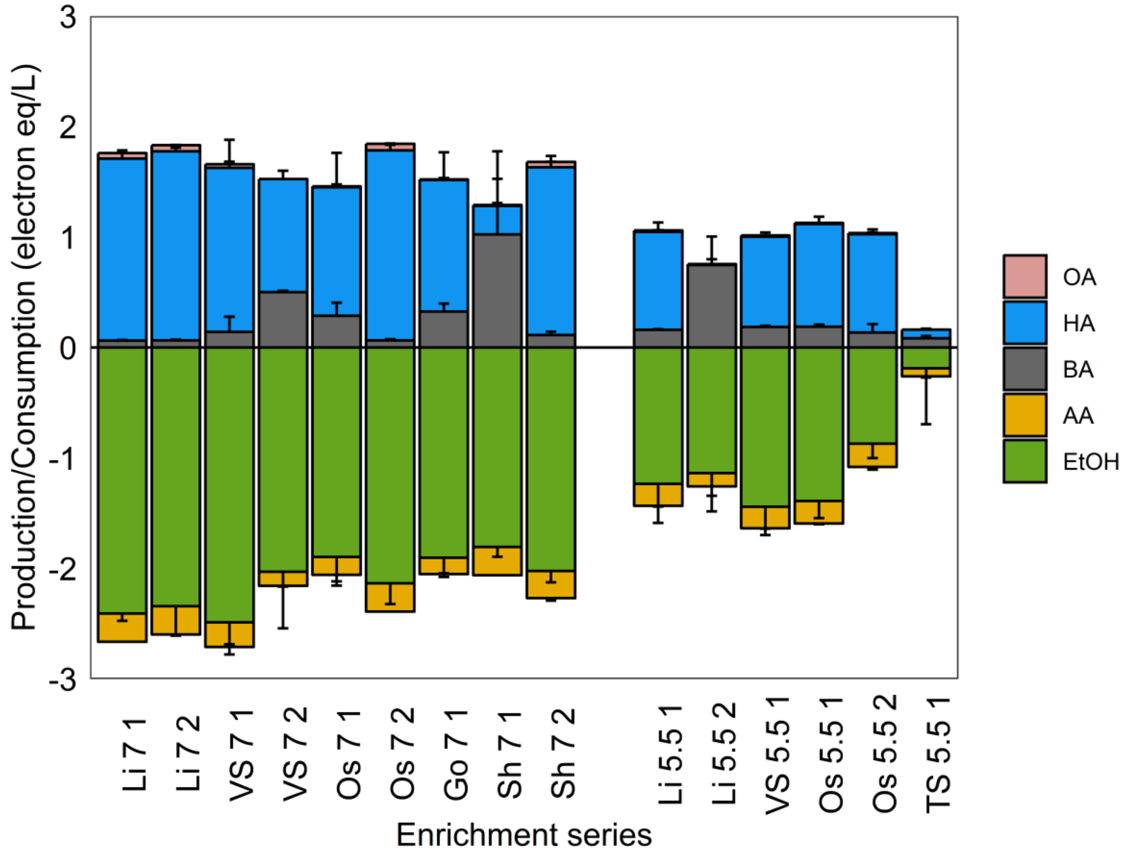

Figure S.14. Electron balance for each enrichment for the growth curve experiment. Error bars indicate standard deviation over all replicates (n=3-5). Sample names were constructed of inoculum origin, enrichment pH and replicate number, e.g. Li 7 1 represents replicate 1 of Lindemans inoculum enriched at pH 7.

S.8. Methods

S.8.1. Medium Composition

Medium for enrichments was modified from the DSM52 medium used for *C. kluyveri*, with a medium composition (in 1L basal medium): 0.31 g K_2HPO_4, 0.23 g KH_2PO_4, 0.25 g NH_4Cl, 0.05 g $MgSO_4.7H_2O$, 0.06 g $MgCl_2$, 2.50 g $NaHCO_3$, 4.88 g 2-(N-morpholino)ethanesulfonic acid (MES), 5.28 g of 2-bromoethanesulfonate (BES), 4.28 g K-Ac and 15 mL ethanol·L^{-1}. Vitamins and trace elements were added: 1 mL of trace element solution SL-10, 1 mL of selenite-tungstate solution and 0.1 mL of 7-vitamin solution. The SL-10 trace element solution consisted of (in 1L stock solution): 10 mL 7.7M HCl, 1.5 g $FeCl_2.4H_2O$, 0.07 g $ZnCl_2$, 0.15 g $MnCl_2.4H_2O$, 0.006 g H_3BO_3, 0.19 g $CoCl_2.6H_2O$, 0.002 g $CuCl_2.2H_2O$, 0.024 g $NiCl_2.6H_2O$

and 0.036 g $Na_2MoO_4.4H_2O$. The selenite-tungstate solution contained (in 1L stock solution): 0.5 g NaOH, 0.003g $Na_2SeO_3.5H_2O$ and 0.004 g $Na_2WO_4.2H_2O$. The 7-vitamin solution consisted of (in 100 mL stock solution): 0.1 g vitamin B12, 0.08 g p-aminobenzoic acid, 0.02 g D(+)-Biotin, 0.2 g nicotinic acid, 0.1 g Ca-pantothenate, 0.3 g pyridoxine hydrochloride, 0.2 g thiamine-HCl.$2H_2O$. Medium without ethanol was sparged for 15 min with N_2/CO_2 (90:10), after which ethanol was added, and pH corrected to 7 using HCl or NaOH. Medium was distributed in 40 mL aliquots in 120 mL serum flasks. Medium was then corrected to pH 5.5 using HCl and again distributed in 40 mL aliquots in 120 mL serum flasks. The headspace was then replaced by N_2 using a gas exchange apparatus. For revival of the communities and subsequent growth curve tests, medium without ethanol, $NaHCO_3$, vitamins and trace elements was first distributed in Balch tubes, and autoclaved for 20 min at 121°C. Subsequently, the necessary additions were supplemented from sterile anaerobic stocks and pH corrected with sterile anaerobic 1M HCl or 1M NaOH. Final working volume was 9 mL per Balch tube (V_{tot} = 26 mL), to maintain the same headspace-liquid ratio.

S.8.2. Enrichment strategy

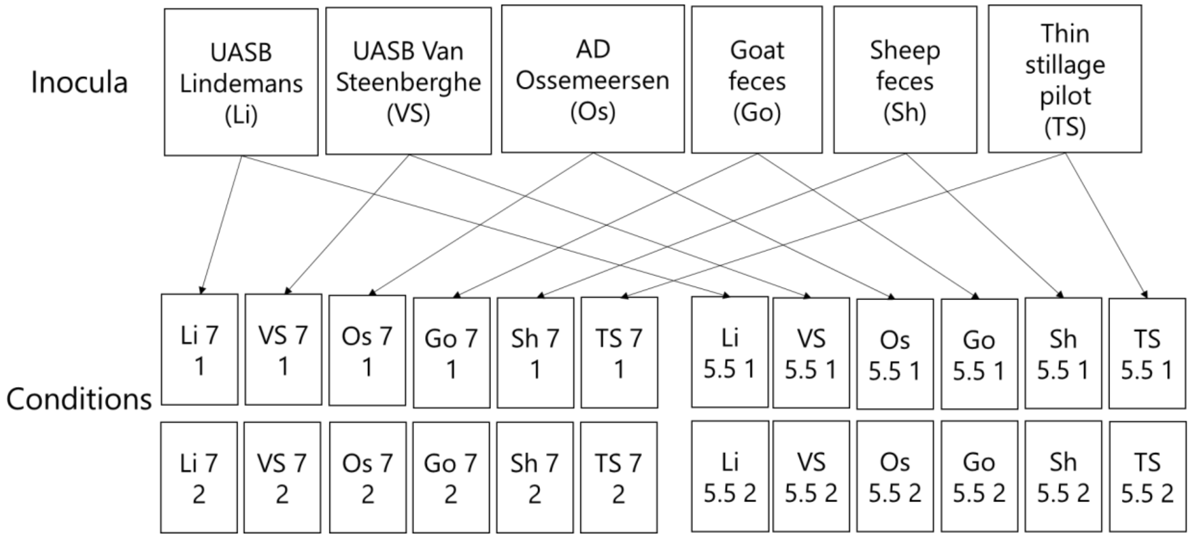

Figure S.15. Schematic overview of enrichment strategy from six inocula, at two pH in duplicate

S.8.3. Chemical analyses

C2 to C8 carboxylic acids (including isoforms C4 to C6) were determined by gas chromatography (GC; GC-2014, Shimadzu®, The Netherlands), with a DB-FFAP 123-3232 column (30 m × 0.32 mm × 0.25 µm; Agilent, Belgium) and a flame ionisation detector (FID). Liquid samples were conditioned with 2 mL sulfuric acid, 200 mg sodium chloride, and 2-methyl hexanoic acid as an internal standard for quantification before further extraction with diethyl ether (1:1 volume sample:ether). The sample (1 µL) was injected at 250°C with a split ratio of 50 and a purge flow of 3 $mL \cdot min^{-1}$. The oven temperature increased by 10°$C \cdot min^{-1}$ from 110 to 250°C where it was maintained for 5 min. The FID had a temperature of 300°C. Nitrogen carrier gas was maintained at a flow rate of 2.49 $mL \cdot min^{-1}$. The GC was externally calibrated with a minimum detection limit of 60 $mg \cdot L^{-1}$ for acetic acid, and 10 $mg \cdot L^{-1}$ for all higher carboxylic acids.

Alcohols (acetone, methanol, ethanol, 1-propanol, 2-propanol, 1-butanol, 1-pentanol and 1-hexanol) were measured by GC (GC-2010-Plus, Shimadzu®, Belgium) with a Stabilwax-DE-S column (30m x 0.32mm x 1.0 µm, Shimadzu®, Belgium) and an FID. 1 µL of sample

was injected at 230°C with a split ratio of 50 and purge flow of 3.0 mL·min^{-1}. Oven temperature was put at 35°C for one minute, after which it increased from 35°C to 230°C at a rate of 10°C min^{-1} where it was kept for 2 minutes. FID temperature was set at 250°C, carrier gas was nitrogen gas at a flow rate of 1.09 mL·min^{-1}. The GC was externally calibrated with a minimum detection limit of 25 mg·L^{-1}.

The gas-phase composition was analyzed with a Compact GC (Global Analyser Solutions, Breda, The Netherlands). CH_4, O_2 and N_2 were analysed with a Molsieve 5A precolumn and Porabond column using He as a carrier gas, H_2 was analysed with the same precolumn and column, but using N_2 as a carrier gas. CO_2, N_2O, and H_2S were analysed using an Rt-Q-bond precolumn and column with He as a carrier gas. A thermal conductivity detector was used to determine gases in the headspace.

S.9. Quality control of sequencing

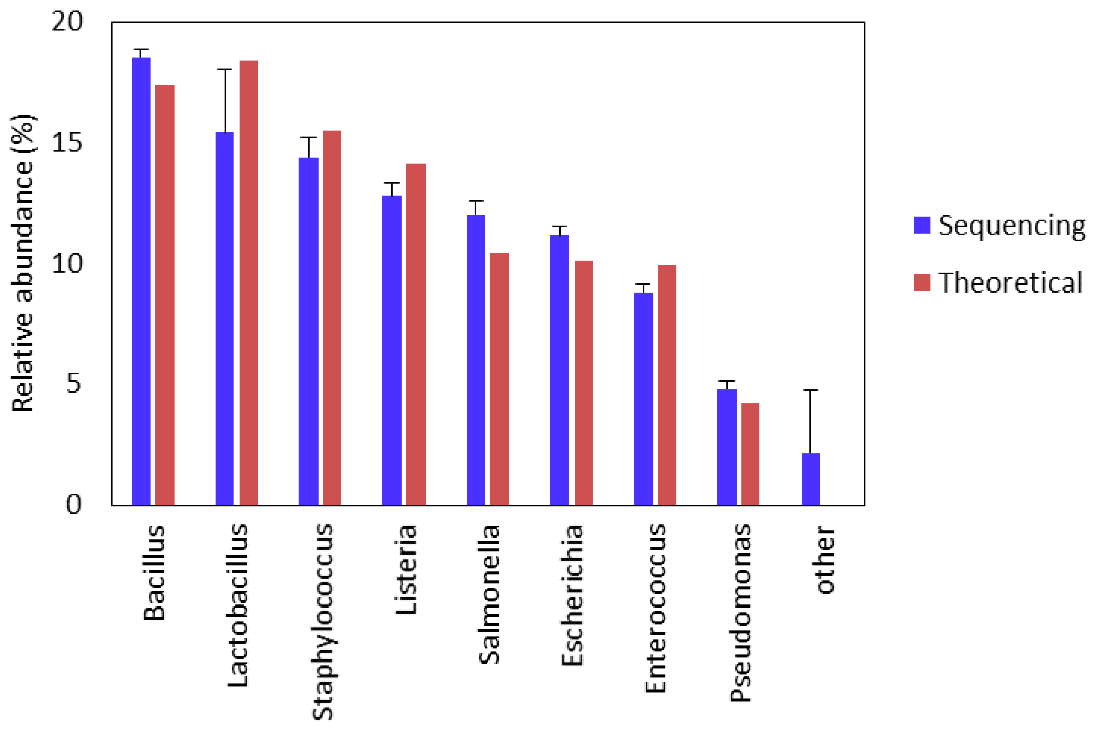

Figure S.16. Quality control of Illumina sequencing by comparison of a mock community with known composition to actual sequencing results, performed in triplicate

8. References

1. Ondov, B. D., Bergman, N. H. & Phillippy, A. M. Interactive metagenomic visualization in a Web browser. *BMC Bioinformatics* **12**, 1–9 (2011).
